# Folate Supplementation Awareness Among Women of Reproductive Age in Poland: Focus on Active Forms and Updated National Recommendations

**DOI:** 10.3390/nu17243881

**Published:** 2025-12-12

**Authors:** Olga Barbarska, Lidia Zaryczny-Trojan, Anna Minkiewicz-Zochniak

**Affiliations:** 1 School of Medical & Health Sciences, VIZJA University, 59 Okopowa St., 01-043 Warsaw, Poland; lidzia.11@interia.pl; 2 Department of Medical Biology, Medical University of Warsaw, Litewska 14/16, 00-575 Warsaw, Poland; anna.minkiewicz@wum.edu.pl

**Keywords:** folic acid, 5-methyltetrahydrofolate, folate supplementation, preconception care, MTHFR, infertility, reproductive health, neural tube defects

## Abstract

**Background:** Adequate folate intake before and during early pregnancy is essential for neural tube defect (NTD) prevention. In 2023, the Polish Society of Gynecologists and Obstetricians updated national recommendations, emphasizing the use of L-5-methyltetrahydrofolate (5-MTHF). Evidence on women’s awareness of these guidelines is limited. **Methods:** This cross-sectional online survey (2025) included 188 Polish women aged 18–45 years who were currently pregnant or had been pregnant within the previous 12 months. Knowledge, attitudes, and supplementation practices were compared between women with (*n* = 94) and without infertility (*n* = 94). Group differences were assessed using χ^2^ tests, and predictors of 5-MTHF use were examined with multivariable logistic regression. **Results:** General awareness of folate recommendations was high (98.9%). However, detailed knowledge varied substantially. Women with infertility more frequently recognized different folate forms (87.2% vs. 72.3%; *p* = 0.018), knew the role of the MTHFR gene (77.7% vs. 39.4%; *p* < 0.001), and initiated supplementation ≥3 months before conception (88.3% vs. 47.9%; *p* < 0.001). Overall, 66% reported using 5-MTHF. Independent predictors of choosing 5-MTHF included awareness of folate forms, MTHFR knowledge, and awareness of emerging considerations related to folic acid metabolism. Infertility status was not an independent predictor. **Conclusions:** Although folate supplementation was nearly universal in this selective sample, more advanced knowledge—particularly regarding folate forms and genetic aspects of folate metabolism—remained limited. Higher awareness among women with infertility likely reflects greater exposure to medical supervision rather than inherent differences between groups. These findings may represent early signals of how the 2023 Polish recommendations are disseminating among women who are more engaged with health information, highlighting the need for continued public and professional education to support informed use of folate supplements.

## 1. Introduction

Folate is essential for one-carbon metabolism, DNA synthesis, methylation, and cell division, making it critical for reproductive health and fetal development [1]. As a key substrate in the folate cycle, it supports remethylation of homocysteine to methionine and contributes to S-adenosylmethionine (SAM) production the universal methyl donor required for epigenetic regulation and tissue differentiation in early pregnancy [2]. Deficiency during pregnancy has been linked to serious maternal and fetal complications, including megaloblastic anemia, preeclampsia, recurrent miscarriage, preterm birth, and especially neural tube defects (NTDs) that develop in the first weeks of gestation [3,4]. Classic randomized controlled trials demonstrated that folic acid supplementation before and during early pregnancy reduces the risk of NTD recurrence [5], and subsequent systematic and narrative reviews confirmed its effectiveness in primary prevention [6,7,8]. Evidence from prospective cohort studies also suggests that adequate maternal folate status during pregnancy may be associated with a lower risk of emotional and behavioural problems in offspring [9]. These findings support the broader concept that maternal micronutrient status may shape neurodevelopmental trajectories via epigenetic mechanisms.

Despite proven benefits, a significant proportion of the population has a limited capacity to metabolize synthetic folic acid due to low activity of dihydrofolate reductase (DHFR) and polymorphisms in the methylenetetrahydrofolate reductase (MTHFR) gene, such as 677C > T. This may contribute to reduced tissue folate and to the presence of unmetabolized folic acid (UMFA), an emerging biochemical finding whose clinical significance remains uncertain [8,10]. Clinical studies have shown that supplementation with L-5-methyltetrahydrofolate (5-MTHF), the biologically active form, achieves comparable–and in some studies slightly higher-increases in red blood cell folate and greater homocysteine reduction, particularly in women with MTHFR variants [11,12,13]. Unlike folic acid, 5-MTHF bypasses the DHFR-dependent reduction steps and directly enters the methylation pathway, which may support improved folate utilization in individuals with reduced enzymatic activity, although current evidence does not establish clear clinical superiority across all outcomes [14]. Moreover, observational data indicate that 5-MTHF is the predominant and more stable circulating form of folate during pregnancy, both in maternal and cord blood, suggesting differences in how 5-MTHF functions physiologically compared with synthetic folic acid [15]. Supplementation with 5-MTHF has also been associated with lower circulating UMFA levels [16], although current evidence does not establish clear clinical implications of UMFA presence at recommended intake levels [8]. The possibility that folic acid might mask hematological signs of vitamin B12 deficiency has been discussed in the literature, but this concern relates primarily to pharmacological doses rather than routine prenatal supplementation, and available evidence remains limited and inconclusive [8].

Recent evidence also indicates that supplementation with 5-MTHF, especially when combined with vitamins B12 and B6, has been explored in relation to reproductive outcomes, including oocyte quality, fertilization success, and live birth rates in women undergoing assisted reproductive technologies (ART) [17]. These findings underscore the broader reproductive relevance of folate metabolism and its potential role in supporting female fertility, particularly in women with infertility or subfertility.

International guidelines consistently recommend supplemental folate at 400 µg/day for women of reproductive age to prevent neural tube defects, as endorsed by the World Health Organization (WHO) and the Centers for Disease Control and Prevention (CDC) [18,19]. The European Food Safety Authority (EFSA) has also recognized that increasing maternal folate status through supplemental intake reduces the risk of neural tube defects, specifying that 400 µg/day of supplemental folate—most commonly as folic acid—should be consumed for at least one month before and up to three months after conception [20].

Similar recommendations are issued by U.S. professional bodies such as the American College of Obstetricians and Gynecologists (ACOG) and the American Academy of Pediatrics (AAP), which recommend 400 µg/day [21,22], while the U.S. Preventive Services Task Force advises a broader range of 400–800 µg/day for all persons planning or able to become pregnant [23].

In contrast, the Polish Society of Gynecologists and Obstetricians issued updated national recommendations in 2023, which prioritize the biologically active form of folate—L-5-methyltetrahydrofolate (5-MTHF). The guidelines advise combined supplementation of 400 µg folic acid and 400 µg 5-MTHF during the periconceptional period, while 5-MTHF alone is preferred during pregnancy and lactation at a dose of 800 µg/day. In women at high risk—such as those with obesity, low folate status, or a previous history of neural tube defects—doses up to 5 mg/day may be recommended [24]. Although the EFSA-authorized health claim “reduces the risk of neural tube defects” formally applies only to synthetic folic acid at 400 µg/day, the scientific opinion acknowledges the comparable biological efficacy of authorized folate forms, including 5-MTHF, in improving maternal folate status and supporting fetal development [20].

In light of the updated Polish recommendations emphasizing active folate forms, it is important to understand how well these changes are reflected in women’s knowledge and supplementation behaviours. As infertility treatment often involves more intensive preconception counselling, women with infertility may differ in awareness and adherence to these guidelines. This study therefore aimed to evaluate knowledge, attitudes, and practices related to folate supplementation among women of reproductive age in Poland, with particular attention to differences between women with and without infertility and to awareness of the new recommendations highlighting 5-MTHF.

## 2. Materials and Methods

This cross-sectional, anonymous online survey was conducted in 2025, among 188 women aged 18–45 years who were either pregnant or had been pregnant within the previous year. Participants were recruited through social media platforms and fertility clinics. The inclusion criteria were: age 18–45 years, confirmed pregnancy or delivery within the past 12 months, and voluntary consent to participate. Respondents were divided into two subgroups: women with a history of infertility (*n* = 94) and women without fertility problems (*n* = 94).

In this study, “infertility” was defined according to the World Health Organization (WHO) as a disease of the reproductive system characterized by the failure to achieve a clinical pregnancy after 12 months of regular unprotected sexual intercourse [25]. This definition was used to classify participants into the infertility and non-infertility groups.

Data were collected using a self-administered questionnaire consisting of 26 closed and semi-open questions. The instrument assessed sociodemographic characteristics, awareness of pregnancy supplementation guidelines, knowledge and use of folate forms (folic acid vs. 5-MTHF) and awareness of the MTHFR gene. The questionnaire was fully anonymous and did not collect any personal identifiers such as name, e-mail address, or IP data. Participation was voluntary, and respondents could withdraw at any time without providing a reason.

Prior to data collection, the questionnaire underwent pilot testing among 12 women of reproductive age to assess clarity, comprehensibility, and the logical flow of items. Based on participant feedback, minor linguistic adjustments were made.

As the study had an exploratory design, no formal a priori power calculation was performed. The final sample size (*n* = 188), including equal numbers of women with and without infertility, reflects all eligible respondents recruited during the study period and was sufficient for the descriptive and comparative analyses conducted.

Statistical analyses were performed using IBM SPSS Statistics version 29.0 (IBM Corp., Armonk, NY, USA) and Python 3.11 (Python Software Foundation, Wilmington, DE, USA) implemented in the Google Colab environment.

Descriptive statistics were used to summarize categorical variables. Between-group differences were assessed using the chi-square (χ^2^) test, with *p* < 0.05 considered statistically significant.

Logistic regression was applied to identify independent predictors of 5-MTHF use. Multivariable logistic regression models were constructed adjusting for demographic, reproductive, knowledge-based, and informational variables. Results were expressed as odds ratios (OR) with 95% confidence intervals (CI). Forest plots visualizing adjusted ORs and 95% CIs were generated in Python using the matplotlib and pandas libraries. A *p*-value < 0.05 was considered statistically significant.

The study was conducted in accordance with the principles of the Declaration of Helsinki. It involved no intervention or collection of identifiable data; therefore, formal ethical approval was not required under national regulations for anonymous, non-interventional studies. Completion and electronic submission of the questionnaire were considered to constitute informed consent to participate in the study and for the collected data to be used for research purposes.

## 3. Results

### 3.1. Sociodemographic Characteristics

A total of 188 women aged 18–45 years were included in the analysis. Most participants were 26–35 years old (75.0%), followed by women aged 18–25-year- (12.2%) and 36–45-year- (12.8%). Women with infertility problems were significantly more likely to belong to the oldest age group (25.5% vs. 8.5% among those without such difficulties), whereas women without infertility issues more frequently represented the 26–35 age category (78.7% vs. 62.8%; *p* = 0.008). The youngest age group showed comparable proportions in both groups.

No significant differences were observed in education level or place of residence between women with and without infertility problems (*p* > 0.05 for both). In the overall sample, 56.4% held a Master’s or PhD degree, 19.1% had a Bachelor’s/engineering degree, and 24.5% reported secondary or vocational education. Regarding residence, 36.2% lived in rural areas, 12.8% in small towns, 22.9% in medium-sized towns, and 28.2% in large cities.

### 3.2. Baseline Knowledge and Practices Related to Folate Supplementation Among Women with and Without Infertility

Baseline differences in folate-related knowledge and supplementation practices between women with and without infertility are summarized in Table 1.

Overall awareness of nutrients recommended during pregnancy was high across the entire sample (98.4%), and nearly all participants reported using folate/folic acid supplements during pregnancy (98.9%), with no significant differences between groups.

However, women with infertility demonstrated higher awareness of specific aspects of folate metabolism. They more frequently reported awareness of different folate forms available in supplements (87.2% vs. 72.3%, *p* = 0.018), recognized methylated folate (5-MTHF) (77.7% vs. 55.3%, *p* = 0.002), and declared understanding of differences between folate forms (59.6% vs. 38.3%, *p* = 0.005). They were also significantly more likely to be familiar with the role of the MTHFR gene in folate metabolism (77.7% vs. 39.4%, *p* < 0.001).

Differences were also observed in preconception practices. Women with infertility were more likely to initiate supplementation at least three months before conception (88.3% vs. 47.9%, *p* < 0.001) and to use the active folate form (5-MTHF) (66.0% vs. 44.7%, *p* = 0.005).

### 3.3. Multivariable Analysis of Predictors of 5-MTHF Use

**Figure 1:**
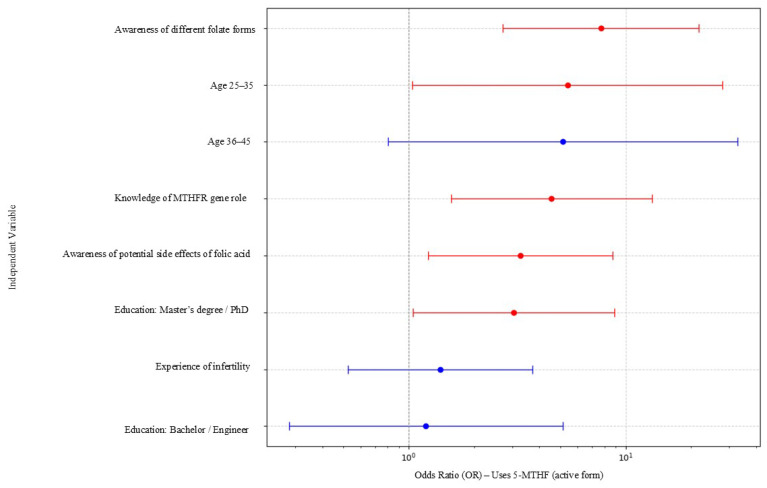
Multivariable logistic regression assessing factors associated with the use of the active folate form (5MTHF) (*n* = 188). Adjusted odds ratios (ORs) with 95% confidence intervals for predictors of using the active folate form (5-MTHF). Significant predictors (*p* < 0.05) are shown in red and non-significant predictors in blue. The vertical dashed line indicates OR = 1.

In the multivariable logistic regression model, several factors were independently associated with the use of the active folate form (5-MTHF) (Figure 1). The strongest predictors included awareness of different folate forms, knowledge of the MTHFR gene, and awareness of potential side effects of folic acid, each showing substantially increased odds of choosing 5-MTHF. Higher educational attainment (Master’s/PhD) and age 25–35 years were also significant positive predictors.

In contrast, age 36–45 years, experience of infertility, and lower educational levels (Bachelor/Engineer) were not significantly associated with the use of 5-MTHF, with confidence intervals crossing 1.

Table 2 summarizes the adjusted odds ratios for factors associated with choosing 5-MTHF. Awareness of different folate forms, knowledge of the MTHFR gene, and awareness of potential side effects of folic acid showed the strongest associations.

## 4. Discussion

This study showed substantial differences in folate-related knowledge and preconception practices between women with and without infertility, although infertility itself was not an independent predictor of 5-MTHF use in the multivariable model. This indicates that the unadjusted differences observed between groups were driven primarily higher knowledge levels and greater exposure to specialist reproductive care among women with infertility, rather than by infertility status alone.

General awareness of folate supplementation during pregnancy was very high in our cohort (98.9%). Women with infertility more frequently recognized different folate forms, including 5-MTHF (77.7% vs. 55.3%, *p* = 0.002), and were significantly more likely to initiate supplementation at least three months before conception (88.3% vs. 47.9%, *p* < 0.001). These findings may reflect increased engagement with reproductive healthcare services and more frequent nutritional counselling. However, detailed understanding of differences between folate forms and the metabolic role of the *MTHFR* gene remained limited across the entire sample, highlighting persistent gaps in more advanced folate-related knowledge. It should be noted that MTHFR-related knowledge in this study reflected only general awareness of the gene and its relevance to folate metabolism, rather than knowledge of personal genotype.

More than half of the respondents lived in medium- or large-sized cities, and most had higher education, which likely contributed to the uniformly high general awareness observed in our study. Importantly, the recruitment strategy—based on social media and fertility clinics—creates a major selection bias toward women who are more educated, health-conscious, digitally active, and engaged with healthcare services. This means that the sample does not reflect the general population of reproductive-age women in Poland and likely overestimates true levels of knowledge and adherence. Sociodemographic factors—particularly education—likely influenced several knowledge-related outcomes. Although education did not consistently predict 5-MTHF use in the multivariable model, adjusting for it attenuated the strength of some associations, indicating a potential confounding effect. Comparable demographic patterns have been reported in national Polish data. In a cross-sectional survey of 2543 women, Kwapień et al. [26] found that adherence to folic acid recommendations was highest women aged 26–35 years and among those with higher education, while younger and less-educated women demonstrated poorer adherence. Similarly, Sobek et al. [27] reported that although folic acid supplementation was common (83.3%), only 28% of women initiated it before pregnancy, with physician recommendation identified as the strongest motivator.

International evidence reinforces the role of socioeconomic and educational gradients in shaping adherence to folate recommendations. In the EuroPrevall birth cohort, folic acid use during pregnancy varied widely—from 55.6% in Lithuania to 97.8% in Spain—and was consistently higher among older and better-educated women [28]. In Ethiopia, only 47.7% of pregnant women adhered to iron–folate supplementation guidelines, with education, knowledge level, and urban residence emerging as significant predictors of compliance [29]. Likewise, in the Philippines, merely 25.8% of women completed the recommended 180-day course of iron and folic acid supplementation [30]. These findings indicate that, despite the high awareness observed in our relatively health-conscious sample, substantial global inequalities persist in access to information and adherence to supplementation practices.

Although folate supplementation was nearly universal, adherence to optimal preconception timing was notably lower. Only 68% of participants began supplementation at least three months before conception, with adherence was particularly low among women without infertility (47.9%). Previous research has shown that women undergoing infertility treatment tend to be more engaged in preconception health behaviours, including micronutrient supplementation [31,32].

Adequate folate status has been associated with improved reproductive outcomes, including ovulatory function, implantation, and overall pregnancy success [17,33]. Evidence from the EARTH cohort links higher supplemental folate intake with more favourable ovarian reserve parameters and improved ART outcomes, especially at doses of 800–1200 μg/day [33]. Furthermore, women undergoing assisted reproductive technology who received 5-MTHF together with vitamins B_12_ and B_6_ showed higher pregnancy and live-birth rates in some studies, compared with those supplemented with folic acid alone [17]. However, these associations should be interpreted cautiously, as available evidence remains heterogeneous and does not allow firm conclusions regarding the comparative effectiveness of specific folate forms in fertility settings.

Our study did not measure fertility endpoints or clinical outcomes; therefore, references to ART-related evidence serve only to contextualize why women with infertility may show higher supplementation awareness and more intensive preconception behaviours. In this context, women experiencing infertility may be more motivated to explore a wide range of evidence-based strategies—including dietary, lifestyle, and supplement-related approaches—which could partly explain their greater interest in different folate forms, including 5-MTHF.

Such behaviours highlight the importance of ensuring equitable, high-quality preconception counselling for all women of reproductive age, not only those receiving specialist fertility care. It is also important to note that major international guidelines continue to recommend synthetic folic acid (400 µg/day) as the standard form for neural tube defect prevention, as endorsed by WHO, CDC, EFSA, ACOG and AAP [18,19,20,21,22,23].

A notable observation in this study is the disconnect between knowledge and behaviour. Despite near-universal awareness of folate recommendations, adherence to optimal preconception timing remained inconsistent unless reinforced by structured medical oversight. This pattern is consistent with findings by Ren et al. [34], who showed that reduced self-control and low information-source credibility hinder the translation of health knowledge into consistent behaviours.

The relatively high proportion of women who recognized 5-MTHF (66% overall) is a promising indicator of the growing reach of updated public and professional messaging in Poland. 5-MTHF represents the biologically active form of folate, and clinical studies have shown that it increases folate status at least as effectively as folic acid, with some evidence suggesting potential benefits in specific subgroups such as individuals with reduced MTHFR activity [13,35]. Evidence from controlled trials demonstrates that both forms effectively improve folate markers, although responses may vary by genotype. Observational data also suggest that 5-MTHF may be associated with differences in circulating UMFA levels; however, the clinical implications of UMFA remain uncertain [16].

Given that MTHFR polymorphisms (C677T and A1298C) occur in up to half of the Caucasian population and are associated with reduced enzyme activity and altered folate–homocysteine metabolism [36], these findings underscore the importance of educating both women and clinicians about the characteristics of different folate forms. In this context, the use of 5-MTHF is sometimes considered a convenient option when an individual’s MTHFR status is unknown, as it can be metabolized independently of MTHFR activity; however, this should not be interpreted as eliminating the potential value of individualized approaches when clinically indicated.

The level of 5-MTHF awareness observed in our sample likely reflects the early public-health impact of the 2023 recommendations of the Polish Society of Gynecologists and Obstetricians, which advise combined supplementation with folic acid and 5-MTHF during the periconceptional period and recommend 5-MTHF alone during pregnancy and lactation, while still permitting the use of synthetic folic acid [24].

Overall, the findings of this study indicate that while folate supplementation is nearly universal within this selective, highly educated sample of Polish women, understanding of form-specific and genetic aspects of folate metabolism remains limited. The growing recognition of 5-MTHF may reflect emerging awareness of alternative folate options as well as the early public-health impact of the 2023 recommendations of the Polish Society of Gynecologists and Obstetricians, which give greater prominence to the active folate form. However, globally endorsed guidelines (WHO, CDC, EFSA, ACOG, AAP) continue to recommend synthetic folic acid as the standard form for neural tube defect prevention, and available evidence does not support strong claims of superiority of 5-MTHF across clinical outcomes. Further research is needed to clarify the comparative effectiveness of different folate forms in both general and high-risk populations.

The study has several limitations. First, its cross-sectional design does not allow determination the directionality of the observed associations. Second, the data were self-reported and not verified against medical or biochemical records, which may introduce recall or reporting bias. Third, the recruitment strategy—based on social media platforms and fertility clinics—likely resulted in substantial selection bias, yielding a sample that overrepresented highly educated, health-conscious, and digitally active women. As a result, both knowledge levels and adherence to supplementation recommendations observed in this study may be considerably higher than in the general population of reproductive-age women in Poland. In addition, the 1:1 distribution of women with and without infertility reflects an intentional sampling strategy designed to enable subgroup comparisons; this proportion does not correspond to population prevalence and therefore limits generalisability of descriptive estimates. Fourth, no a priori power calculation was performed due to the exploratory nature of the study, which may limit the precision of subgroup comparisons. Despite these limitations, the study provides valuable insight into early public awareness of active folate forms and reflects the initial public-health impact of the 2023 recommendations of the Polish Society of Gynecologists and Obstetricians.

## 5. Conclusions

In this survey of 188 pregnant and recently pregnant women, overall awareness of folate supplementation was very high, but detailed understanding of folate forms and the metabolic role of MTHFR remained limited. Women with a history of infertility reported higher awareness and earlier initiation of supplementation, likely reflecting more intensive medical supervision rather than inherent differences between groups.

Use of 5-MTHF was mainly associated with knowledge-related factors such as awareness of folate forms and familiarity with MTHFR, together with higher education levels, underscoring the role of health literacy in shaping supplementation choices. Despite widespread awareness, optimal preconception timing of supplementation was not consistently achieved.

Because the sample was selective—well educated, digitally active, and partly recruited through fertility clinics—the findings cannot be generalized to the entire population of reproductive-age women in Poland. However, the relatively high recognition of 5-MTHF in this subgroup may represent an early behavioural signal of how the updated 2023 national recommendations are beginning to disseminate among women who are more engaged with health information.

Major international guidelines (WHO, CDC, EFSA, ACOG, AAP) continue to recommend synthetic folic acid as the standard for neural tube defect prevention. Current evidence shows that both folic acid and 5-MTHF effectively improve folate status, while comparative clinical advantages remain uncertain. Differences in national recommendations reflect varying interpretations of biochemical considerations and evolving evidence, which explains why the recent Polish guideline update places stronger emphasis on 5-MTHF. Continued public and professional education will be important to support informed, evidence-based use of folate supplements within the context of differing national and international recommendations.

## Figures and Tables

**Table 1:** Comparison of folate-related knowledge and supplementation practices between women with and without infertility. Statistically significant differences (*p* < 0.05) are indicated with an asterisk (*).

Variable	Total (*n* = 188)	Without Infertility (*n* = 94)	With Infertility (*n* = 94)	*p*-Value	χ^2^
Knowledge of nutrients recommended during pregnancy	185 (98.4%)	91 (96.8%)	94 (100.0%)	0.244	1.35
Familiarity with folate/folic acid supplementation recommendations for pregnancy	165 (87.8%)	80 (85.1%)	85 (90.4%)	0.373	0.79
Awareness of different folate forms available in supplements	150 (79.8%)	68 (72.3%)	82 (87.2%)	0.018	5.57
Recognition of methylated folate (5-MTHF)	125 (66.5%)	52 (55.3%)	73 (77.7%)	0.002 *	9.55
Understanding of differences between folate forms	92 (48.9%)	36 (38.3%)	56 (59.6%)	0.005 *	7.68
Knowledge of the MTHFR gene and its role in folate metabolism	110 (58.5%)	37 (39.4%)	73 (77.7%)	<=0.001 *	26.84
Exposure to information about potential adverse effects of synthetic folic acid	80 (42.6%)	37 (39.4%)	43 (45.7%)	0.460	0.54
Reported use of folate/folic acid supplements during pregnancy	186 (98.9%)	92 (97.9%)	94 (100.0%)	0.477	0.51
Initiation of supplementation ≥3 months before conception	128 (68.1%)	45 (47.9%)	83 (88.3%)	<0.001 *	33.51
Use of the active folate form (5-MTHF)	104 (55.3%)	42 (44.7%)	62 (66.0%)	0.005 *	7.77
Choosing supplements based on specialist recommendations	136 (72.3%)	69 (73.4%)	67 (71.3%)	0.870	0.03
Self-rated knowledge: good/very good	124 (65.9%)	56 (59.6%)	68 (72.3%)	0.090	2.87

**Table 2:** Multivariable logistic regression analysis of factors associated with the use of the active folate form (5-MTHF) (*n* = 188), with adjusted odds ratios (ORs) and 95% confidence intervals (CI).

Variable	Odds Ratio (OR)	95% CI Lower	95% CI Upper
Awareness of different folate forms	6.79	1.40	32.82
Knowledge of MTHFR gene role	3.22	1.28	8.10
Awareness of potential side effects of folic acid	2.72	1.19	6.24
Education: Master’s degree/PhD	3.02	1.05	8.65
Age 25–35	4.59	1.18	17.79
Age 36–45	3.27	0.74	14.42
Education: Bachelor/Engineer	1.43	0.36	5.64
Experience of infertility	1.21	0.52	2.83

## Data Availability

The data underlying this article are available from the corresponding author upon reasonable request.

## Abbreviations

AAP	American Academy of Pediatrics
ACOG	American College of Obstetricians and Gynecologists
ART	Assisted Reproductive Technologies
CDC	Centers for Disease Control and Prevention
CI	Confidence Interval
DHFR	Dihydrofolate Reductase
EFSA	European Food Safety Authority
MTHFR	Methylenetetrahydrofolate Reductase
NTD	Neural Tube Defect
OR	Odds Ratio
USPSTF	U.S. Preventive Services Task Force
UMFA	Unmetabolized Folic Acid
WHO	World Health Organization
5-MTHF	5-methyltetrahydrofolate
